# A rare combination of gastroschisis, jejunal atresia, colonic atresia, and midgut malrotation in a newborn: a case report and literature review

**DOI:** 10.1097/RC9.0000000000000755

**Published:** 2026-07-30

**Authors:** Layth J.M. Saada, Malak Ismael Marei, Tareq Fiqyat, Yaman Abu Sarrees, Amir Atawneh, Jamil Saada

**Affiliations:** a Department of Surgery, Al-Mezan Speciality Hospital, Hebron, Palestine; b Faculty of Medicine and Health Sciences, Al-Quds University, Jerusalem, Palestine; c Faculty of Medicine and Health Sciences, An-Najah National University, Nablus, Palestine; d Pediatric Intensive Care Unit, Department of Pediatrics, Al-Mezan Speciality Hospital, Hebron, Palestine; e Department of Pediatric Surgery, Al-Quds University, Jerusalem, Palestine; f Department of Pediatric Surgery, Al-Mezan Speciality Hospital, Hebron, Palestine

**Keywords:** case report, colonic atresia, gastroschisis, jejunal atresia, malrotation, neonatal surgery

## Abstract

**Introduction and importance:**

Gastroschisis is a congenital full-thickness anterior abdominal wall defect that allows bowel evisceration without a covering sac. While most cases are isolated and have good outcomes, complex gastroschisis involves associated intestinal pathology and carries significantly higher morbidity and mortality. The simultaneous occurrence of jejunal atresia, colonic atresia, and midgut malrotation in the setting of gastroschisis is extraordinarily rare.

**Case presentation:**

A male infant was delivered at 36 weeks’ gestation following an antenatal diagnosis of gastroschisis. At birth, marked abdominal distension and bilious nasogastric aspirate raised immediate concern for an associated anomaly. Intraoperative assessment revealed type IIIb jejunal atresia, type I right colonic atresia, midgut malrotation, and an unused microcolon. The colonic atresia was only identified after further inspection to ensure distal bowel patency. Single-stage repair was performed, consisting of primary jejunoileal and colocolic anastomoses, Ladd’s procedure, and abdominal wall closure. The early postoperative course was encouraging, with full oral feeding achieved by the second postoperative week. However, by week 6, the infant developed medical necrotizing enterocolitis, septic shock due to an ESBL-producing Klebsiella, and multiorgan failure, and died on day 48 of life.

**Clinical discussion:**

Complex gastroschisis with multiple gastrointestinal anomalies severely limits intestinal function, increases susceptibility to infection, and complicates operative and postoperative recovery. Even following a technically successful repair, optimizing nutrition and preventing sepsis remain difficult.

**Conclusion:**

Careful intraoperative distal bowel evaluation is essential to avoid missed atresias. Mortality remains notable in this exceedingly rare combination due to the cumulative burden of underlying anomalies and infectious complications.

## Introduction

Gastroschisis, the most common type of congenital abdominal wall defect, is a paraumbilical full-thickness defect of the anterior abdominal wall and is most commonly located to the right of the umbilicus, allowing evisceration of bowel loops without a covering sac^[^1^]^. Over recent decades, the incidence of gastroschisis has been rising, estimated at 4 per 10 000 live births, and remains closely associated with young maternal age^[^1–3^]^. Most cases of gastroschisis range from simple gastroschisis, which is an isolated evisceration with functional bowel, to complex gastroschisis, which accounts for 15–17% of cases and is defined by associated intestinal compromise such as atresia, necrosis, perforation, or volvulus, and shows higher morbidity, longer hospitalization, and greater nutritional requirements^[^3^]^.

HIGHLIGHTS

- Complex gastroschisis with jejunal–colonic atresias and midgut malrotation is exceptionally rare and clinically challenging.
- Abdominal distension at birth in neonates with gastroschisis should raise suspicion for hidden intestinal anomalies to prevent a delayed diagnosis.
- Intraoperative verification of distal bowel patency is essential, as multiple atresias may coexist and otherwise remain unrecognized.
- Despite optimal repair, survival is not always achieved; timely nutritional support and strict infection control are vital for recovery in these fragile neonates.
- Reporting such rare cases enhances awareness and informs better surgical decision-making and postoperative management.

In 10–20% of cases, complex gastroschisis is associated with intestinal atresia and is considered the most frequent associated anomaly, most commonly in the small bowel (jejunoileal)^[^3–5^]^. However, colonic atresia in association with gastroschisis is extremely rare and, when present, often poses diagnostic and therapeutic challenges^[^6^]^. Reports describing gastroschisis, jejunal atresia, and colonic atresia in the same patient are rare but instructive, with only a handful of cases reported worldwide^[^7–9^]^. This complex association reflects severe intrauterine vascular disruption and often illustrates the diagnostic and surgical complexity, emphasizing the need for careful intraoperative inspection and management^[^10,11^]^.

In this report, we present a rare case of gastroschisis associated with both jejunal and colonic atresias, as well as concurrent midgut malrotation, highlighting the diagnostic challenges, operative management, and postoperative outcome in the context of the previously reported literature. This report follows the SCARE 2025 criteria^[^12^]^.

## Case presentation

**Figure 1.**
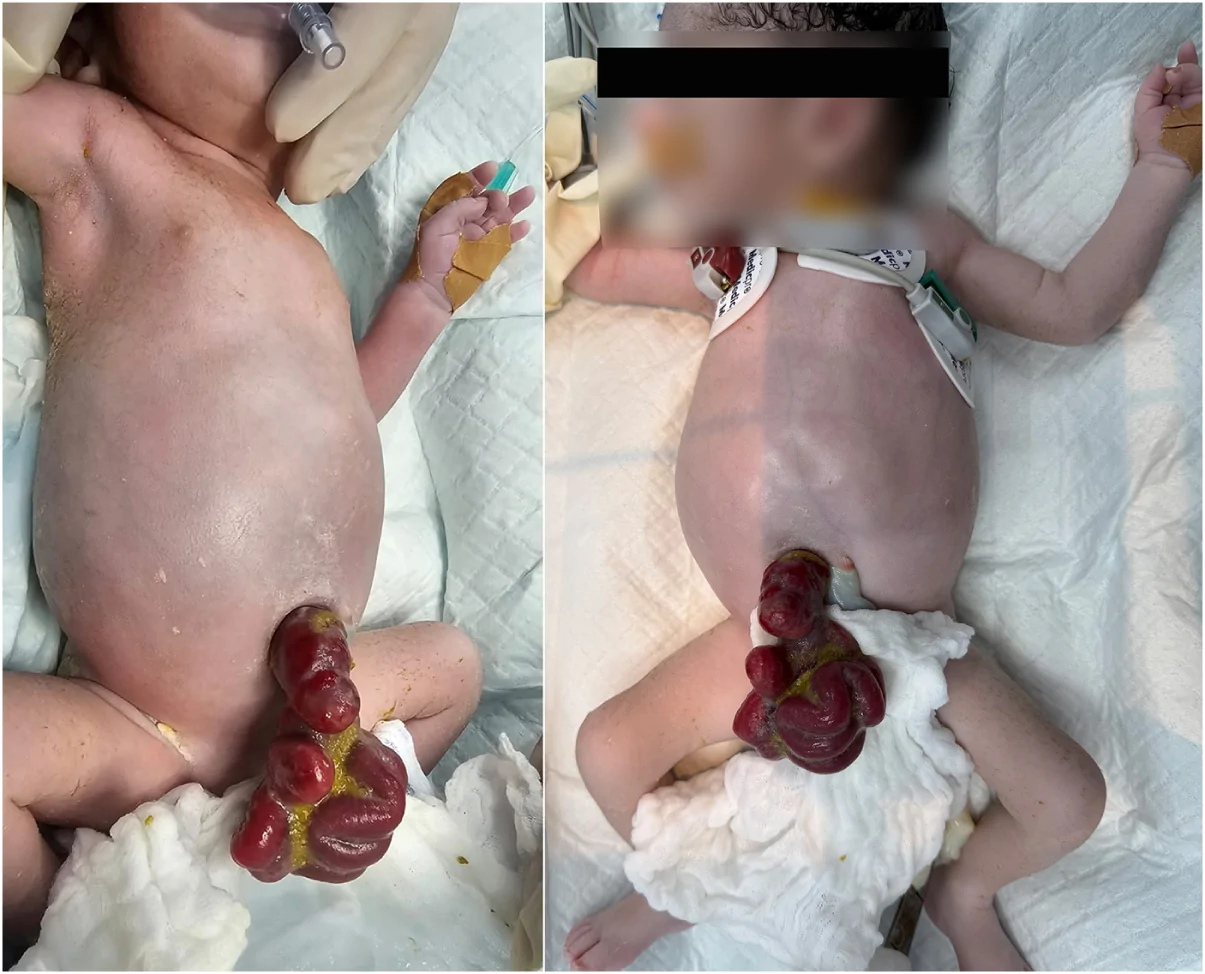
Immediate post-delivery photograph showing a paraumbilical abdominal wall defect with exteriorized, meconium-stained bowel loops, consistent with gastroschisis, along with marked abdominal distension.

A male neonate was delivered by cesarean section at 36 weeks’ gestation to a 20-year-old mother (G2P2) with first-degree consanguinity, following an antenatal diagnosis of gastroschisis at 20 weeks’ gestation. One day before delivery, a single dose of dexamethasone was administered, and the rest of the pregnancy was uneventful. At birth, the infant weighed 2070 g, with Apgar scores of 7 and 8 at 1 and 5 minutes, respectively. Examination revealed a paraumbilical abdominal wall defect with externalized bowel loops, indicative of gastroschisis (Fig. 1).

**Figure 2.**
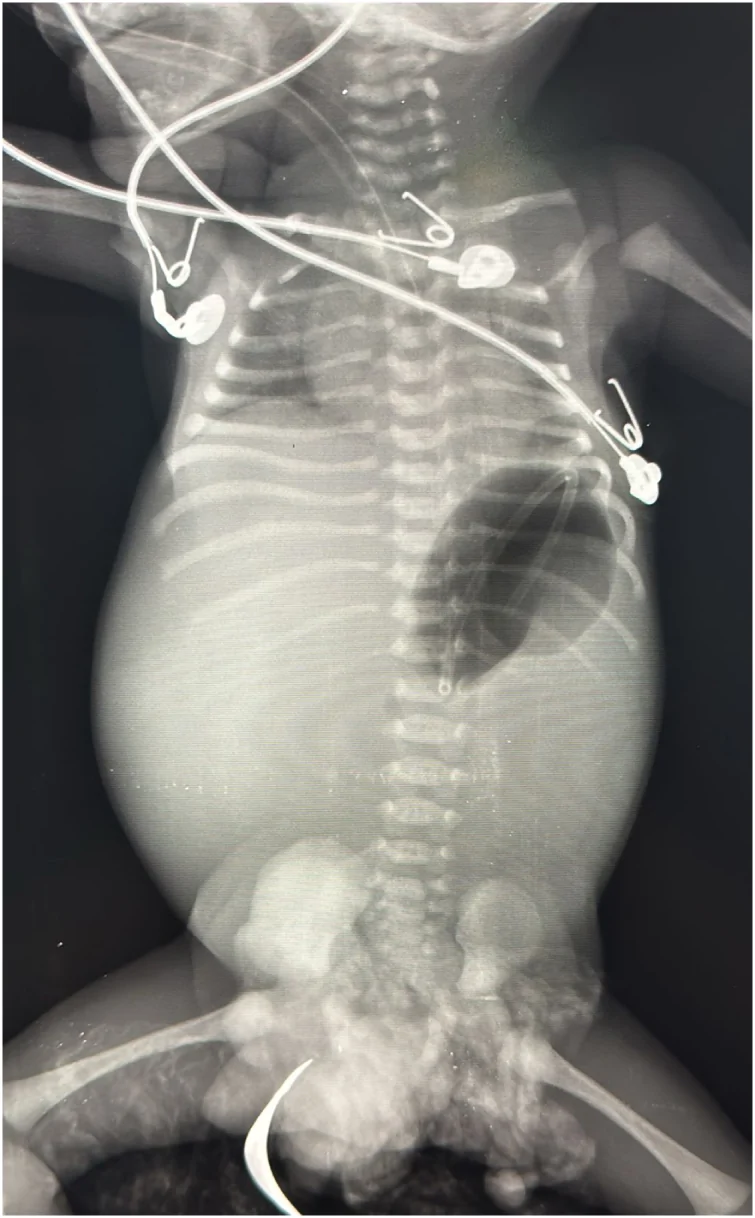
Portable abdominal radiograph demonstrating a gasless abdomen with a single gastric bubble, suggestive of intestinal atresia.

**Figure 3.**
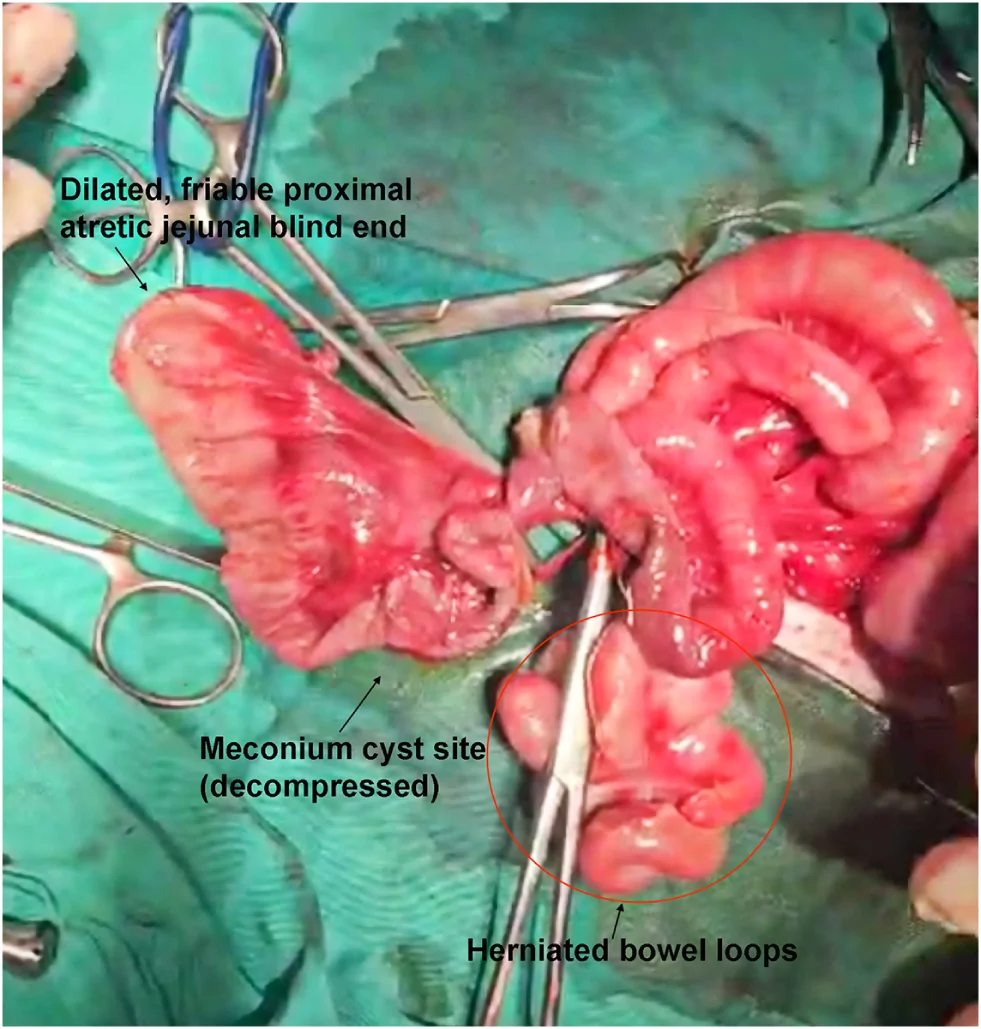
Intraoperative photograph showing a dilated, friable proximal jejunal blind end with a decompressed meconium cyst site and herniated bowel loops.

**Figure 4.**
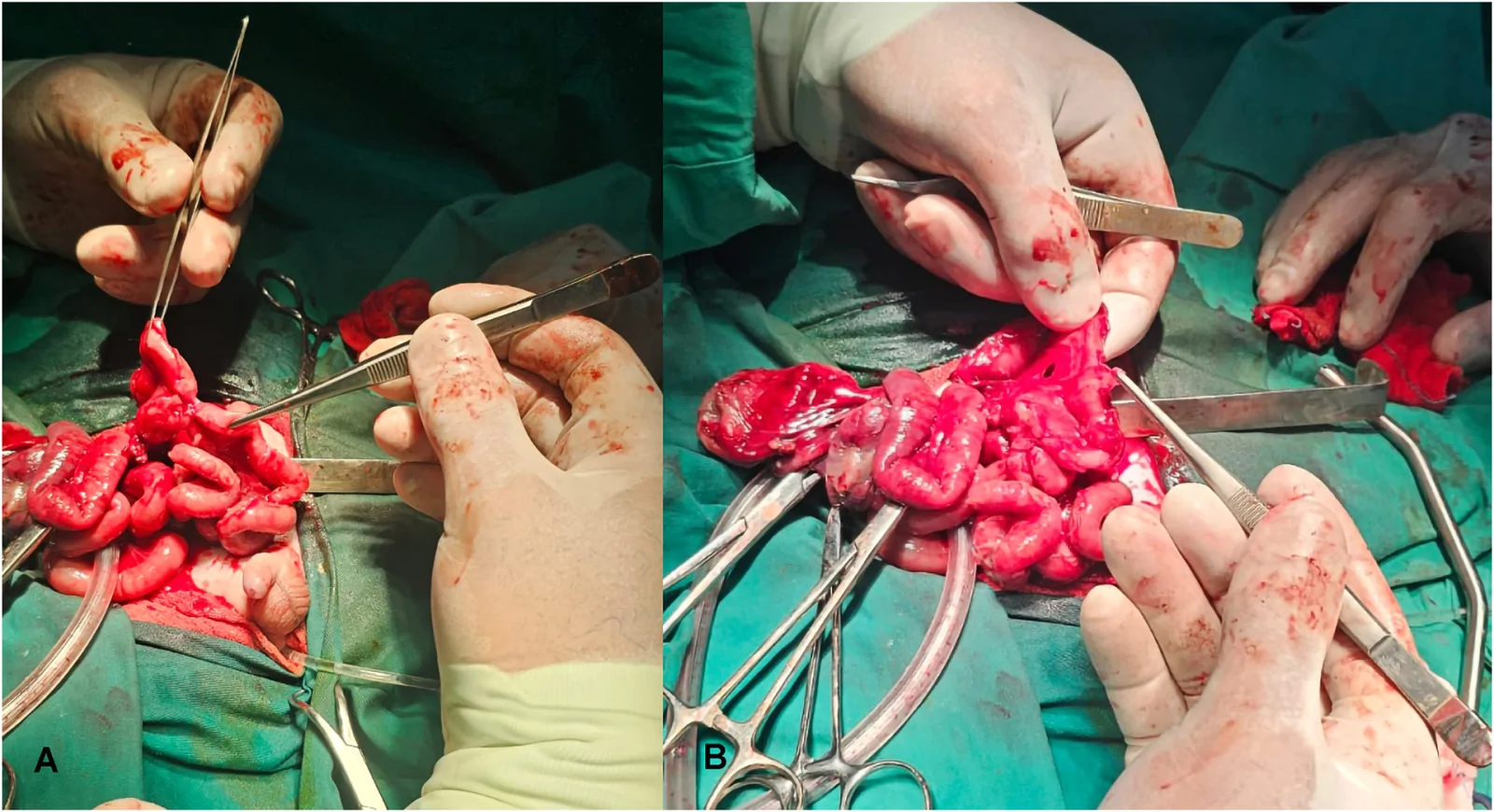
Intraoperative photographs (A and B) showing colonic atresia with an unused microcolon located between the ascending and transverse colon. (A) After enterotomy of the distal blind segment of the small-bowel atresia with saline injection, both the proximal and distal segments adjacent to the colonic atresia became mildly dilated.

Immediately after delivery, the neonate was transported in sterile drapes to a secondary operating theater for a planned primary closure of gastroschisis. Following initial resuscitation, marked abdominal distension was noted (Fig. 1), and nasogastric decompression yielded a large volume of bilious fluid. Strong suspicion of an underlying intestinal atresia was raised by a portable abdominal radiograph that showed a gasless abdomen with a single gastric bubble (Fig. 2). Consequently, the procedure was converted to an exploratory laparotomy by extending the existing gastroschisis defect into a supraumbilical midline incision. Upon delivery of the bowel, multiple grossly distended, fluid-filled, blind-ended bowel loops were identified (Fig. 3; *see* Supplemental Digital Content, Video 1, available at: https://links.lww.com/IJSCR/A99). Enterotomy of the distal blind segment, about 30 cm proximal to the ileocecal valve, followed by saline injection, confirmed an atretic right-colonic segment (Fig. 4). The absence of a duodenal C-loop, with the duodenum coursing entirely to the right side of the abdomen, was consistent with an associated intestinal malrotation.

**Figure 5.**
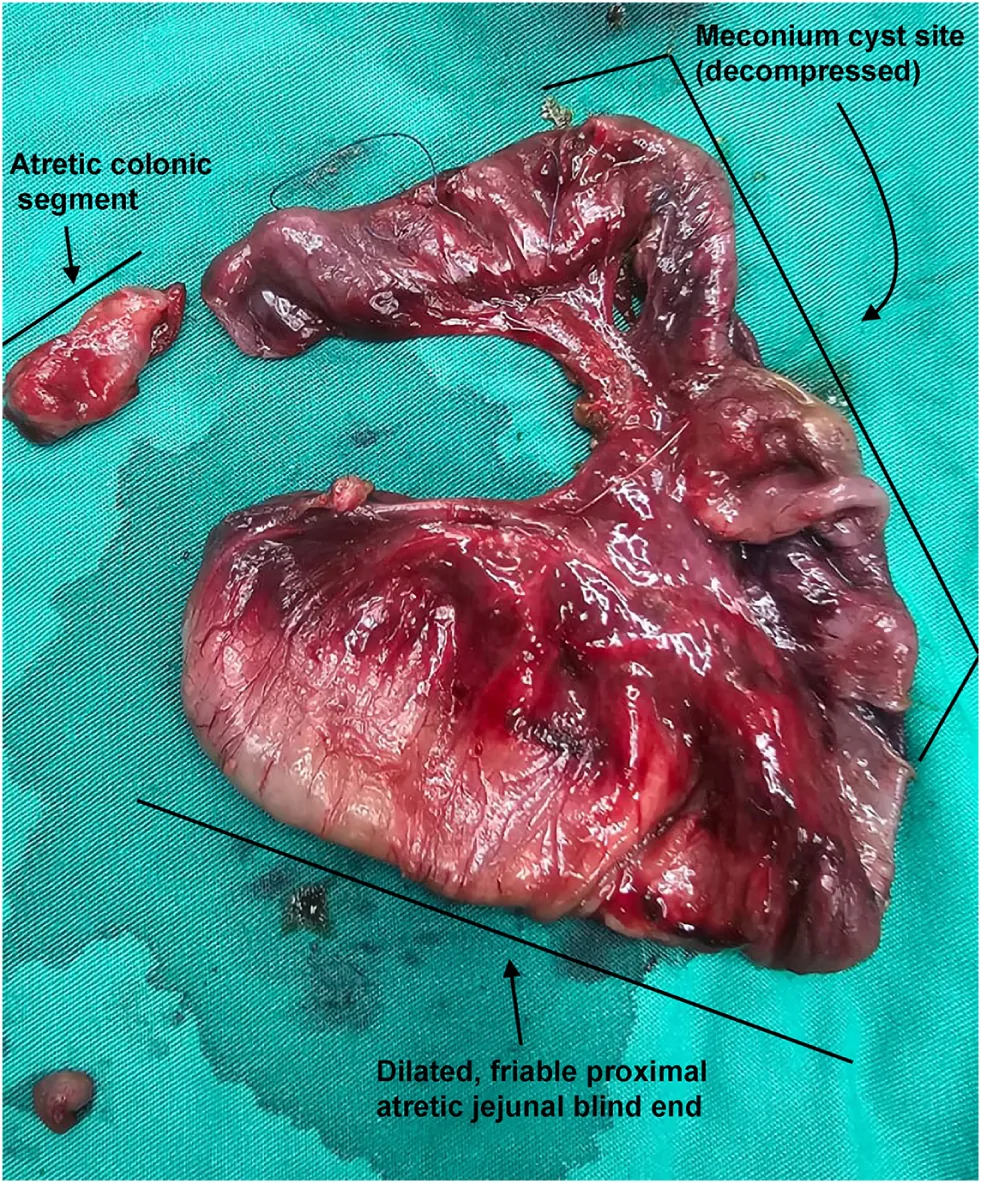
Resected specimen showing a markedly dilated, friable proximal jejunal blind end measuring approximately 20 cm, with the site of a decompressed meconium cyst and a short atretic colonic segment measuring about 1 cm.

**Figure 6.**
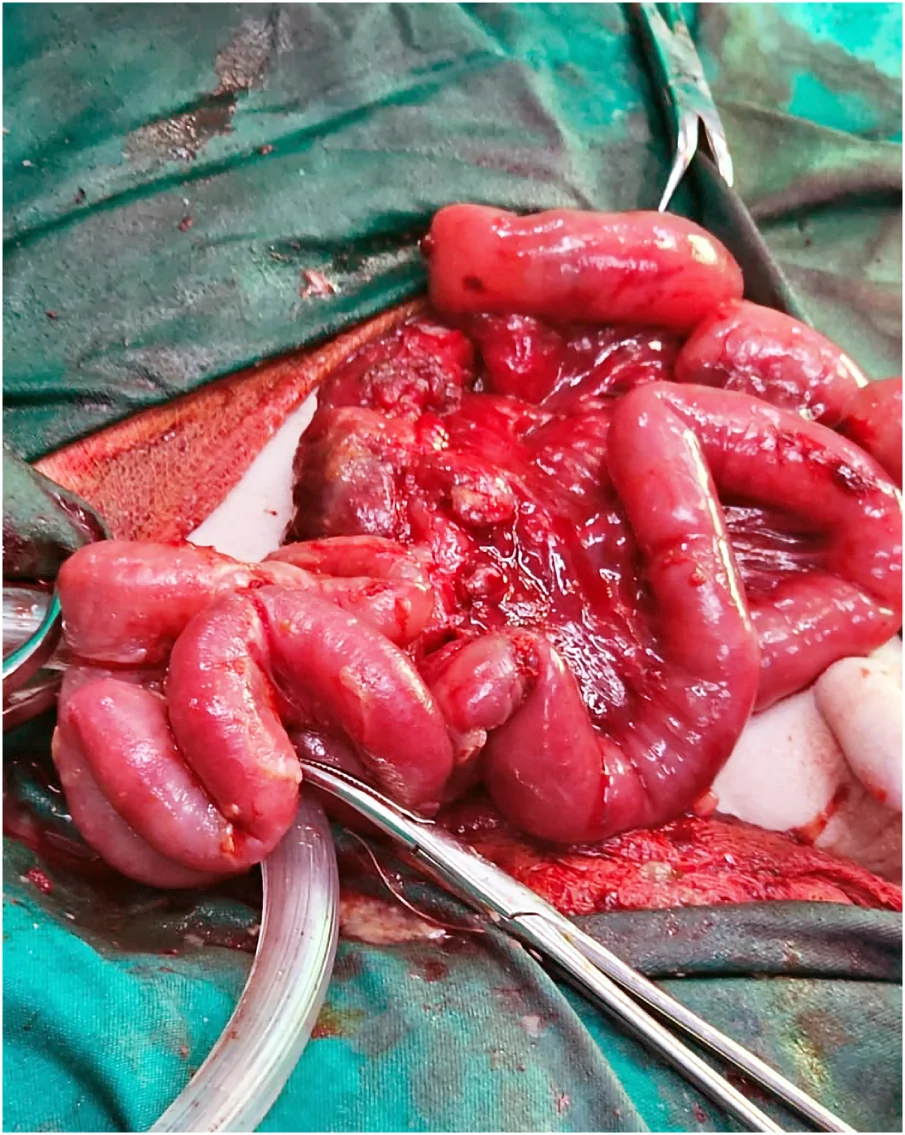
Postoperative photograph demonstrating restored bowel continuity after primary jejuno-ileal and colocolic anastomoses, with mesenteric widening of the herniated bowel.

Operative findings revealed gastroschisis associated with atypical type III jejunal atresia, morphologically closer to type IIIb (coiling of the herniated bowel); type I right colonic atresia; midgut malrotation; and an unused microcolon. Approximately 20 cm of markedly dilated and friable jejunum, ending in the proximal blind pouch of the atresia and including a very dilated meconium cyst, was resected (Fig. 5), followed by a primary jejunoileal anastomosis. The atretic right colonic segment, approximately 1 cm in length, was also resected (Fig. 5), and a primary colocolic anastomosis was performed. Widening of the mesenteric base of the herniated bowel (Ladd’s procedure) was performed, and the bowel was reduced into the abdomen. The postoperative photograph demonstrates the remaining bowel with restored continuity (Fig. 6). After confirming ductal patency, the abdominal wall defect was closed. The patient received packed red blood cells intraoperatively and was transferred, intubated and sedated, to the neonatal intensive care unit.

Postoperatively, the infant was kept nil per os and maintained on parenteral support. He remained ventilated and required inotropes for transient hypotension. On postoperative days 3–4, clonic seizures developed; electroencephalography demonstrated diffuse background suppression, and phenobarbital achieved good control. Laboratory investigations revealed leukocytosis (26 × 10^3^/µL), thrombocytopenia (platelets 135 × 10^3^/µL), and elevated C-reactive protein (CRP 250 mg/L). Blood culture grew *Streptococcus viridans*, confirming early-onset sepsis. Clinical and laboratory results improved after antimicrobial therapy was changed, with normalization of inflammatory markers by postoperative day 10 (WBC 10.9 × 10^3^/µL, CRP 7.7 mg/L). He was successfully extubated on postoperative day 6 and gradually switched to nasal cannula oxygen. Trophic feeds were initiated on day 10 after the passage of bowel movements, were well tolerated, and were progressively advanced to full oral feeding by the end of the second postoperative week.

By weeks 3–4, feeds were advanced to full enteral volumes. The infant achieved modest weight gain with frequent loose stools. Echocardiography at week 3 showed a small patent ductus arteriosus and a foramen ovale, with mild left pulmonary artery stenosis. During the same period, he developed a febrile episode with leukocytosis (22.1 × 10^3^/µL) and elevated CRP (375 mg/L); all cultures were negative, and the episode was treated as clinical sepsis with gradual improvement. During weeks 4–5, CRP stabilized at 20–30 mg/L while leukocytosis (30.0 × 10^3^/µL) recurred. Repeat blood cultures and removal of the central line for tip culture showed no growth, and these were followed by mild, gradual hematologic improvement.

**Figure 7.**
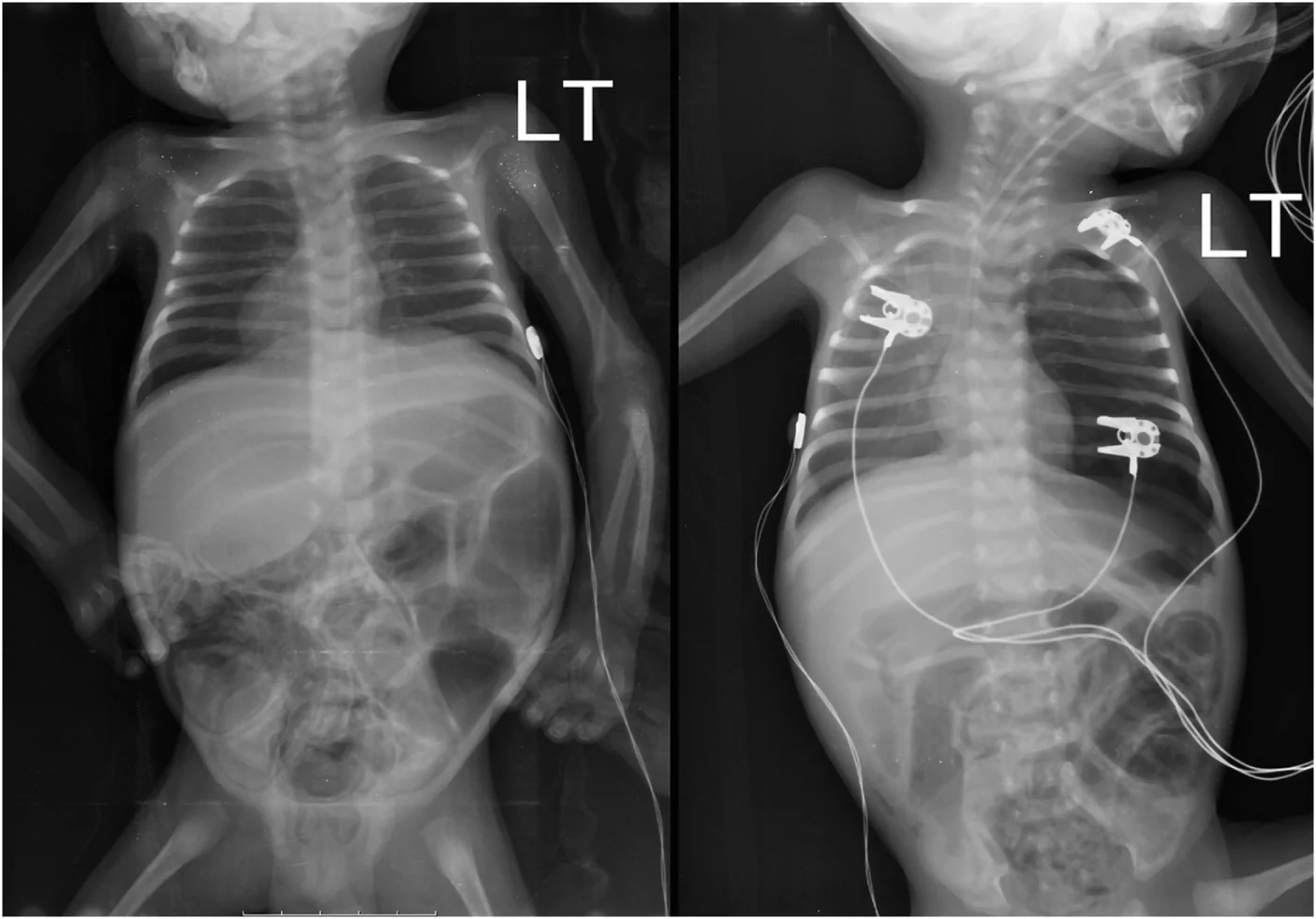
An abdominal radiograph shows pneumatosis intestinalis consistent with necrotizing enterocolitis (Bell stage II) during the sixth postoperative week.

During weeks 5–6, while maintained on full oral feeds, the infant received fortified breast milk and hydrolyzed formula; however, he demonstrated poor weight gain due to frequent loose stools. In week 6, he developed abdominal distension, diarrhea, hematemesis, and lethargy. Abdominal ultrasonography confirmed the findings of imaging, which showed pneumatosis intestinalis consistent with necrotizing enterocolitis (Bell’s stage II; Fig. 7). A full septic workup was performed: a complete blood count showed leukocytosis (WBC 68.0 × 10^3^/µL), thrombocytosis (platelets 565 × 10^3^/µL), and elevated CRP (186 mg/L). Urine culture grew an ESBL-producing *Klebsiella* species resistant to most antibiotics. He was kept nil per os and received antimicrobial and supportive therapy.

Despite close monitoring, the infant’s condition worsened over time, leading to septic shock and multiorgan dysfunction. He required intubation and mechanical ventilation, along with escalation of inotropic and ventilatory support. Laboratory results showed acute kidney injury (creatinine 2.6 mg/dL, BUN 67 mg/dL), coagulopathy, and metabolic acidosis (pH 6.97). Multiple transfusions of packed red blood cells, fresh frozen plasma, vitamin K, and albumin were administered as part of supportive care. The infant suffered three cardiopulmonary arrests in the last days of week 6 and was pronounced dead at 48 days of age despite intensive resuscitation efforts.

## Discussion

We report an exceedingly rare case of complex gastroschisis associated with multiple gastrointestinal anomalies. Unlike previously reported cases in which jejunal and/or colonic atresia occurred with gastroschisis^[^6,8,9^]^, our patient presented with simultaneous type IIIb jejunal atresia and type I right colonic atresia together with midgut malrotation, and was managed with single-stage primary repair at birth. This combination represents one of the rarest forms of complex gastroschisis reported to date. The case highlights the diagnostic and operative challenges and discusses postoperative complications, including necrotizing enterocolitis and recurrent sepsis.

Newborns with the triad of gastroschisis and colonic and jejunal atresias usually face a more severe surgical and postoperative course compared with patients with isolated or simple gastroschisis^[^13,14^]^. These infants often require multiple surgical interventions and/or prolonged stays in the neonatal intensive care unit. Furthermore, prolonged dependence on total parenteral nutrition, delayed initiation of enteral feeding, and an increased risk of short bowel syndrome are also observed^[^15,16^]^.

A summary of previously reported cases of gastroschisis with additional gastrointestinal anomalies (primarily involving two or more) is presented in Table 1, demonstrating heterogeneity in anatomical configurations, management approaches, and postoperative outcomes.

**Table 1:** Summary of previously reported cases of gastroschisis associated with additional gastrointestinal anomalies (mainly two or more), highlighting differences in anatomical configurations, surgical management, and postoperative outcomes.

Study ID	Associated Gastrointestinal Anomalies	Gestational age; Birth weight; Sex	Management	Post-operative Course/Complications	Outcome
Saxonhouse *et al*, 2009^[^8^]^	Twin A: Gastroschisis + jejunal & colonic atresia; Twin B: Isolated colonic atresia leading to cecal perforation	Both M; 31 wk; Twin A: 1543 g; Twin B: 1690 g	Twin A underwent staged management with initial primary reduction and mucus fistula formation followed by delayed primary anastomosis and tapering enteroplasty. Twin B had a single-stage laparotomy with right colectomy and primary ileocolic anastomosis	Twin A: full enteral feeds before discharge; Twin B: full oral feeds by discharge.	Survived
Bauman & Nanagas Jr, 2015^[^7^]^	Gastroschisis + type III proximal jejunal atresia (dx day 27) + type II transverse colonic atresia (dx day 48)	M; 35 wk; 2495 g	Initial primary reduction and abdominal closure for gastroschisis. Later required multiple laparotomies: (1) Day 27: exploratory laparotomy with extensive enterolysis, resection of 8 cm distal atretic jejunum, primary hand-sewn end-to-end enteroenterostomy, and jejunal plication; (2) Day 48: repeat laparotomy with enterolysis and hand-sewn end-to-end anastomosis.	Prolonged ileus with high nasogastric outputs, hyperbilirubinemia secondary to prolonged TPN, electrolyte imbalances, and inadequate weight gain.	Survived
Ashton *et al*, 2014^[^9^]^	Gastroschisis + jejunal & colonic atresias → later developed jejunal stricture at previous anastomosis site.	NR	Neonatally: NR; later exploratory laparotomy for jejunal stricture (spherical trichobezoar was identified proximal to the jejunal stricture).	Post-operative: excellent recovery with complete resolution of edema within 2 weeks; normalization of serum albumin; steady weight and height gain at 5-month follow-up; no further distension or vomiting; remains well on long-term supplements.Note:Preoperative: severe malnutrition, hypoalbuminaemia, oedema, iron deficiency, pancreatic insufficiency (moderate), bacterial overgrowth.	Survived
Shironomae *et al*, 2019^[^17^]^	Gastroschisis + multiple small-intestinal atresias (Type IV: 11 × Type I, 4 × Type II) + impending small-bowel rupture + non-rotation malrotation	F; 35 wk; 1698 g	Emergency laparotomy on day 0: repair of perforation, resection of affected small intestine, creation of separate stoma, primary closure of abdominal wall defect. Stoma closure performed at 68 days of age	Enteral day 8 (stenting tube, mother’s milk); oral day 12.	Survived
Elrouby & Maher, 2021^[^18^]^	Gastroschisis + malrotation + tubular gut duplication + Meckel’s diverticulum	M; full term; BW NR	Exploratory laparotomy with Ladd’s procedure, excision of duplication cyst, Meckel’s diverticulum left in situ, and primary closure of the abdominal wall defect.	Enteral/oral feeds started POD 7, well tolerated. Sepsis, thrombocytopenia, respiratory distress, and hypotension; required mechanical ventilation.	Died in hospital on POD 14 due to profound septic shock.
Pawar *et al*, 2021^[^19^]^	Vanishing (closed/closing) gastroschisis + jejunal atresia + extreme short-bowel syndrome	Case 1: F; 32 wk; 1950 g/Case 2: F; 33 wk; 1500 g	Case 1: Laparotomy → jejunostomy with distal colonic mucus fistula; palliative decision./Case 2: Jejuno-colic end-to-back anastomosis with primary abdominal closure; histology: ischemic bowel necrosis.	Sepsis and septicemic shock in both.	Both died (Case 1: POD 25 at home; Case 2: POD 4 in hospital) due to short-bowel syndrome and sepsis
Darwish *et al*, 2020^[^20^]^	Closing gastroschisis with vanishing midgut syndrome + intra-abdominal volvulus + jejunal atresia + absent ileocecal valve + short bowel syndrome	F; 32 wk; 1900 g	Emergency laparotomy: untwisting of proximal jejunum, excision of necrotic bowel, and tube jejunostomy for drainage and prevention of re-twisting.	Long-term home-based TPN → high risk of intestinal failure syndrome	Survived
Guanà *et al*, 2023^[^21^]^	Vanishing gastroschisis (Type D) + Type IIIA jejunal atresia + extensive midgut atresia + absent terminal ileum & cecum + short bowel syndrome	F; 32 wk, 1600 g	Initial laparotomy: creation of jejunostomy and colostomy (blind ends; small bowel 13 cm and colon 22 cm). At 6 months: jejuno-colonic anastomosis. At 18 months: autologous intestinal lengthening (Bianchi procedure) to achieve autonomy from TPN, with temporary gastrostomy and colostomy.	Partial TPN dependence for 13 months (3 days/week) and mild, improving cholestasis.	Survived
Khalil *et al*, 2010^[^22^]^	Vanishing gastroschisis + short bowel syndrome + absent ileocecal valve + absence of ascending & transverse colon	M; 36 wk; 2530 g	Day 1: Laparotomy → tube jejunostomy + tube colostomy for controlled bowel expansion and colonic recycling. 6 months: Longitudinal Intestinal Lengthening and Tapering (LILT, Bianchi procedure); bowel length increased from 30 cm → 75 cm.	Progressive bowel adaptation and stable recovery. Enteral feeds established post-lengthening, full by 15 months of age.	Survived
Lotakis et al, 2023^[^23^]^	Gastroschisis + type IIIb ileal atresia + ascending colon atresia	F; 35 wk, BW:NR	At birth: the fascia was opened and a silo was placed. Day 7: Early primary anastomoses at the time of abdominal wall closure.	Oral breast milk feeds were initiated on postoperative day 8 at 10cc/kg of breast mild and advanced by 10cc/kg/day.	Survived (12-month follow-up)
Đorđević et al, 2017^[^24^]^	Gastroschisis + colonic atresia	M; 37 wk; 3150 g	Primary reduction of gastroschisis at birth with two-layer end-oblique primary colonic anastomosis followed by primary abdominal wall closure.	Relaparotomy on postoperative day 15 showed kinking proximal to the anastomosis causing mechanical obstruction; adhesiolysis was performed. The patient was discharged home after 32 days.	Survived (4 years follow-up)

BW, birth weight; GA, gestational age; NR, not reported; TPN, total parenteral nutrition.

### Brief embryologic and pathophysiologic mechanisms

Multiple hypotheses have been proposed to explain the pathogenesis of gastroschisis, such as abnormal involution of the right umbilical vein, which leads to impaired viability of the surrounding mesenchyme and failure of embryonic mesenchymal differentiation due to teratogenic exposure^[^3^]^. Complex gastroschisis is most commonly explained by an intrauterine vascular insult affecting mesenteric perfusion^[^3,25,26^]^. Compression at the site of the abdominal wall defect, volvulus of the eviscerated bowel loops, or intramniotic vascular compromise may result in ischemic necrosis of the bowel, atresia formation, bowel shortening, and impaired midgut rotation^[^25–29^]^. In our patient, this mechanism may explain the simultaneous presence of multiple bowel segments as well as a rotational anomaly.

### Diagnostic and intraoperative challenges

Differentiating simple from complex gastroschisis before birth remains a persistent diagnostic challenge. Ultrasound can show bowel dilation outside the abdomen, thickened or echogenic bowel walls, and polyhydramnios, but these signs are not specific to intestinal atresia, necrosis, or malrotation^[^30,31^]^. Therefore, gastroschisis may seem isolated at birth, although hidden distal anomalies can become evident postnatally.

At birth, the presence of marked abdominal distension or a bilious nasogastric aspirate should raise immediate concern for an associated anomaly beyond gastroschisis, such as intestinal atresia. In such cases, prompt abdominal radiography and early surgical assessment are warranted to exclude additional obstructive pathology. In our case, we identified complex gastroschisis immediately after birth, during the initial surgery. Although primary closure was planned, the presence of significant abdominal distension and a bilious nasogastric aspirate raised a strong suspicion of an additional anomaly. An abdominal X-ray taken during surgery showed a gasless abdomen with only a single gastric bubble, which supported the diagnosis of intestinal atresia. The plan was therefore shifted to exploratory laparotomy, which revealed a jejunal atresia; further inspection to ensure distal bowel patency identified an additional colonic atresia. Careful intraoperative evaluation of the entire bowel, including the colon, mesentery, and duodenojejunal junction, is essential in complex gastroschisis to avoid missing distal intestinal anomalies or rotational defects^[^32,33^]^. When bowel assessment is limited by inflammatory peel or staged reduction, exploratory laparotomy may be necessary, particularly when clinical findings raise concern for intestinal obstruction, to confirm distal bowel patency and exclude additional atresias^[^33,34^]^.

Postnatally, when such findings are not evident at birth, ongoing vigilance is essential. Progressive abdominal distension, failure to pass meconium, bilious nasogastric aspirates, or abnormal findings on abdominal radiography should prompt immediate consideration of an underlying atresia^[^3,7,35,36^]^ and early surgical re-evaluation to prevent delays in diagnosis and management, as well as associated morbidity. Delayed recognition of distal atresia after initial gastroschisis closure has previously been reported, including a case described by Bauman and Nanagas Jr. (2015) in which jejunal and colonic atresias were identified at different time points several weeks after initial abdominal wall closure due to persistent symptoms of bowel obstruction^[^7,32^]^.

### Surgical management considerations

Surgical management of complex gastroschisis with multiple intestinal atresias remains controversial, particularly regarding the choice between primary anastomosis and staged diversion. Enterostomy may reduce anastomotic stress in high-risk neonates, while primary repair can preserve intestinal length and avoid stoma-related morbidity^[^37,38^]^.

Although some authors advocate staged repair with enterostomy in complex gastroschisis to reduce anastomotic complications in this fragile population, with favorable outcomes reported following the procedure in some reports^[^6,8,17,20–22^]^, successful primary anastomosis has also been described in the literature. Lotakis *et al*^[23]^ described a patient with complex gastroschisis associated with type IIIb ileal atresia and colonic atresia who underwent early primary anastomoses at the time of abdominal wall closure, resulting in good survival and a favorable 12-month follow-up. Similarly, Đorđević *et al*^[24]^ reported successful primary end-to-end repair in a patient with gastroschisis and colonic atresia and noted survival up to 4 years. In contrast, Pawar *et al*^[^19^]^ reported two infants with complex gastroschisis and jejunal atresia, one treated with enterostomy and the other with primary anastomosis; both died of sepsis and short bowel syndrome.

In our patient, the decision to proceed with resection and primary jejuno-ileal and colo-colic anastomoses was based on good bowel perfusion, minimal luminal size discrepancy, and the desire to preserve intestinal length. The infant initially showed favorable postoperative recovery, achieving full oral feeds by the end of the second postoperative week and maintaining full oral feeds through the fifth postoperative week. However, during the sixth postoperative week, he developed medically managed necrotizing enterocolitis (Bell’s stage II) and recurrent sepsis, and he ultimately survived until day 48 of life. Given the clearly delayed deterioration and the severity of complications, particularly sepsis and necrotizing enterocolitis, these complications were most likely attributable to the natural course of complex gastroschisis and its associated anomalies rather than to the burden of single-stage intestinal reconstruction. This is further supported by the presence of ESBL-producing bacteria and type IIIb jejunal atresia, both of which are associated with significantly higher mortality in this population. Overall, the coexistence of gastroschisis with multiple gastrointestinal anomalies remains exceptionally rare, and the optimal surgical strategy is still debated, as current evidence remains limited to a small number of case reports and isolated experiences.

### Outcomes and prognostic factors

Complex gastroschisis, prematurity, and type IIIb jejunal atresia are associated with delayed intestinal adaptation, feeding intolerance, sepsis, prolonged hospitalization, and increased risk of necrotizing enterocolitis^[^39,40^]^. In a multicenter cohort, infants born at 35–36 weeks had delayed attainment of full enteral feeding and higher rates of medical necrotizing enterocolitis compared with term infants^[^41^]^. In our patient, several factors likely contributed to the later development of necrotizing enterocolitis, including late prematurity (36 weeks), complex gastroschisis, parenteral support, microcolon, and altered intestinal motility associated with malrotation. The presence of type IIIb jejunal atresia likely further worsened the prognosis in our patient, as this severe subtype is associated with extensive bowel loss, prolonged nutritional dependence, and increased mortality compared with other forms of jejunoileal atresia^[^27^]^.

Despite the severity of the underlying anomalies, the infant initially showed an encouraging postoperative recovery. However, poor weight gain, loose stools, recurrent inflammatory episodes, and later abdominal distension preceded clinical deterioration. The patient subsequently developed septic shock, acute kidney injury, and multiorgan failure despite intensive medical management. In our case, an ESBL-producing Klebsiella infection likely contributed to the terminal septic episode and poor outcome because of the limited antimicrobial treatment options and the high mortality associated with these infections^[^42^]^.

Population-based studies have shown that infants with complex gastroschisis are far more likely to develop intestinal failure and bowel obstruction than those with simple gastroschisis, particularly within the first 2 years of life^[^43^]^. Youssef *et al*^[^44^]^ reported that complex gastroschisis was associated with a longer hospital stay and higher mortality compared with simple gastroschisis (9.3% vs 3.4%). Similarly, Bergholz *et al*^[^45^]^ demonstrated significantly higher mortality in complex cases (16.7% vs 2.2%), while Emil *et al*^[^5^]^ identified complex gastroschisis as the single most important predictor of increased morbidity and adverse outcomes. These findings highlight the severe prognostic burden associated with intestinal compromise in complex gastroschisis, particularly in patients with multiple gastrointestinal anomalies, as in our case.

## Conclusion

This case illustrates the severe postoperative challenges associated with the exceptionally rare coexistence of gastroschisis with jejunal and colonic atresias and concurrent malrotation. It also highlights the importance of careful intraoperative evaluation for hidden distal atresias in patients with gastroschisis. Despite technically successful primary repair and an initially encouraging postoperative course, weeks later, the patient developed medical necrotizing enterocolitis, ESBL-producing Klebsiella septic shock, and multi-organ failure. The combination of these factors in the setting of complex gastroschisis likely contributed to the unfavorable outcome observed in this case.

## Data Availability

All data supporting this case report are included in the article, and additional information can be provided upon reasonable request.
